# A Rare Case of Post-sternotomy Mediastinitis Caused by Methicillin-Resistant Staphylococcus pseudintermedius, a Canine Commensal, Following Ascending Aortic Replacement

**DOI:** 10.7759/cureus.86589

**Published:** 2025-06-23

**Authors:** Soichiro Ota, Yuki Hayashi, Atsushi Harada, Naoki Eguchi, Masashi Tanaka

**Affiliations:** 1 Cardiovascular Surgery, Nihon University School of Medicine, Tokyo, JPN

**Keywords:** ascending aortic replacement, cardiac surgery complications, coagulase-positive staphylococci, methicillin-resistant staphylococcus pseudintermedius, multidrug-resistant organisms, pet ownership and infection risk, poststernotomy mediastinitis, surgical site infection, vacuum-assisted closure therapy, zoonotic infection

## Abstract

Post-sternotomy mediastinitis (PSM) is one of the most serious infectious complications following cardiac surgery. It requires prompt diagnosis and comprehensive treatment, including antimicrobial therapy, surgical intervention, and wound management. In recent years, vacuum-assisted closure (VAC) therapy has become a widely accepted strategy for effective infection control and wound healing. It also serves as a valuable bridging therapy before definitive reconstruction, when needed.

We report a rare case of PSM caused by methicillin-resistant *Staphylococcus pseudintermedius* (MRSP), a coagulase-positive Staphylococcus commonly found on dog skin. A 75-year-old man underwent ascending aortic replacement for a thoracic aortic aneurysm and developed fever and wound inflammation on postoperative day 11. Blood and wound cultures confirmed the presence of MRSP. Notably, the patient had no direct contact with the pet dog during hospitalization, suggesting that preoperative skin colonization was a likely source of infection.

The patient underwent debridement, intravenous antibiotics, and VAC therapy. Infection control and wound healing were achieved without flap reconstruction, and the patient was discharged uneventfully. PSM caused by MRSP is extremely rare, and this case highlights the potential role of zoonotic pathogens in postoperative infections.

The findings emphasize the importance of including pet ownership history in preoperative assessments and incorporating it into perioperative infection control planning. Awareness of rare but clinically relevant pathogens, such as MRSP, is essential for the effective management of surgical site infections.

## Introduction

Poststernotomy mediastinitis (PSM) is a serious complication of cardiovascular surgery, and early detection with multidisciplinary management is crucial for improving outcomes. With increasing pet ownership, zoonotic infections caused by *Staphylococcus pseudintermedius* (*S. pseudintermedius*) - a common commensal infection in dogs - have been increasingly reported. Methicillin-resistant *S. pseudintermedius* (MRSP) is an extremely rare but clinically significant pathogen in humans. Unlike *Staphylococcus aureus *(*S. aureus*), which is a well-recognized cause of healthcare-associated infections, *S. pseudintermedius* has traditionally been associated with veterinary infections. However, recent reports have highlighted its potential for colonization and infection in humans, particularly in immunocompromised hosts or those with close contact with companion animals. The emergence of methicillin-resistant strains further complicates management, given their multidrug resistance profiles and diagnostic challenges due to morphological similarity to *S. aureus*.

We report a rare case of PSM caused by MRSP following ascending aortic replacement. This case highlights the importance of considering zoonotic pathogens in the management of surgical infections and the role of comprehensive perioperative assessment.

## Case presentation

A 75-year-old man with hypertension and hyperuricemia was referred to our hospital after a screening examination that revealed dilation of the ascending aorta. Contrast-enhanced computed tomography (CT) revealed a spindle-shaped thoracic aortic aneurysm with a maximum diameter of 50 mm. On admission, his vital signs were stable, and laboratory tests revealed mild renal impairment (creatinine 1.24 mg/dL).

Chest X-ray showed no abnormalities, and transthoracic echocardiography revealed preserved left ventricular systolic function (ejection fraction 68.7%), mild-to-moderate aortic regurgitation, and trivial mitral and tricuspid regurgitation. Coronary CT angiography revealed no significant stenosis, and the patient underwent elective ascending aortic replacement. The operative time was four hours and 39 minutes, cardiopulmonary bypass time was 35 minutes, aortic cross-clamp time was 99 minutes, and circulatory arrest was 25 minutes. Blood transfusions included six units of red blood cells, eight units of fresh frozen plasma, and 20 units of platelets.

**Figure 1:**
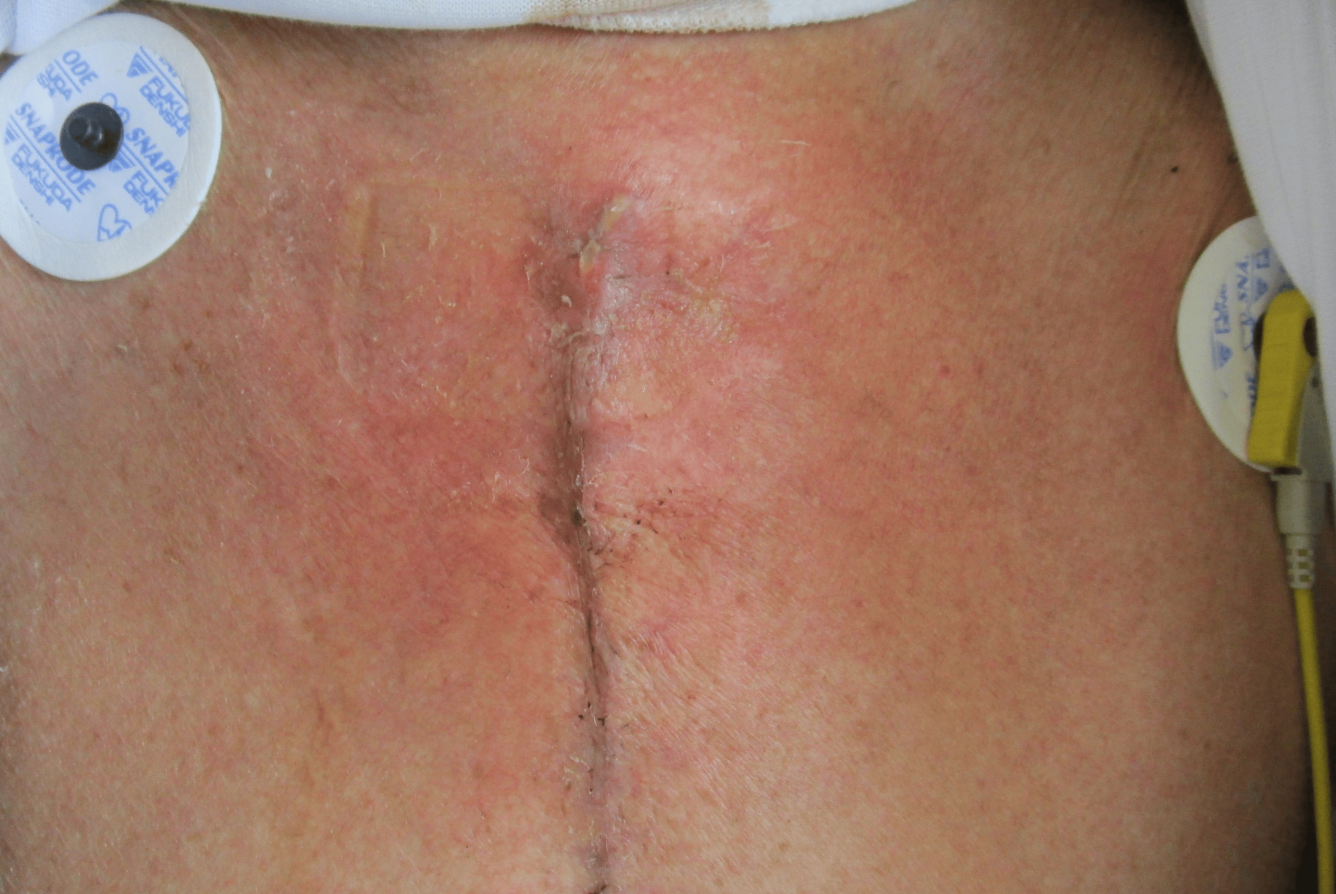
Wound appearance on postoperative day 14 (before incision) Erythema is present around the surgical wound, with yellowish-white purulent discharge from the cranial end of the incision

**Figure 2:**
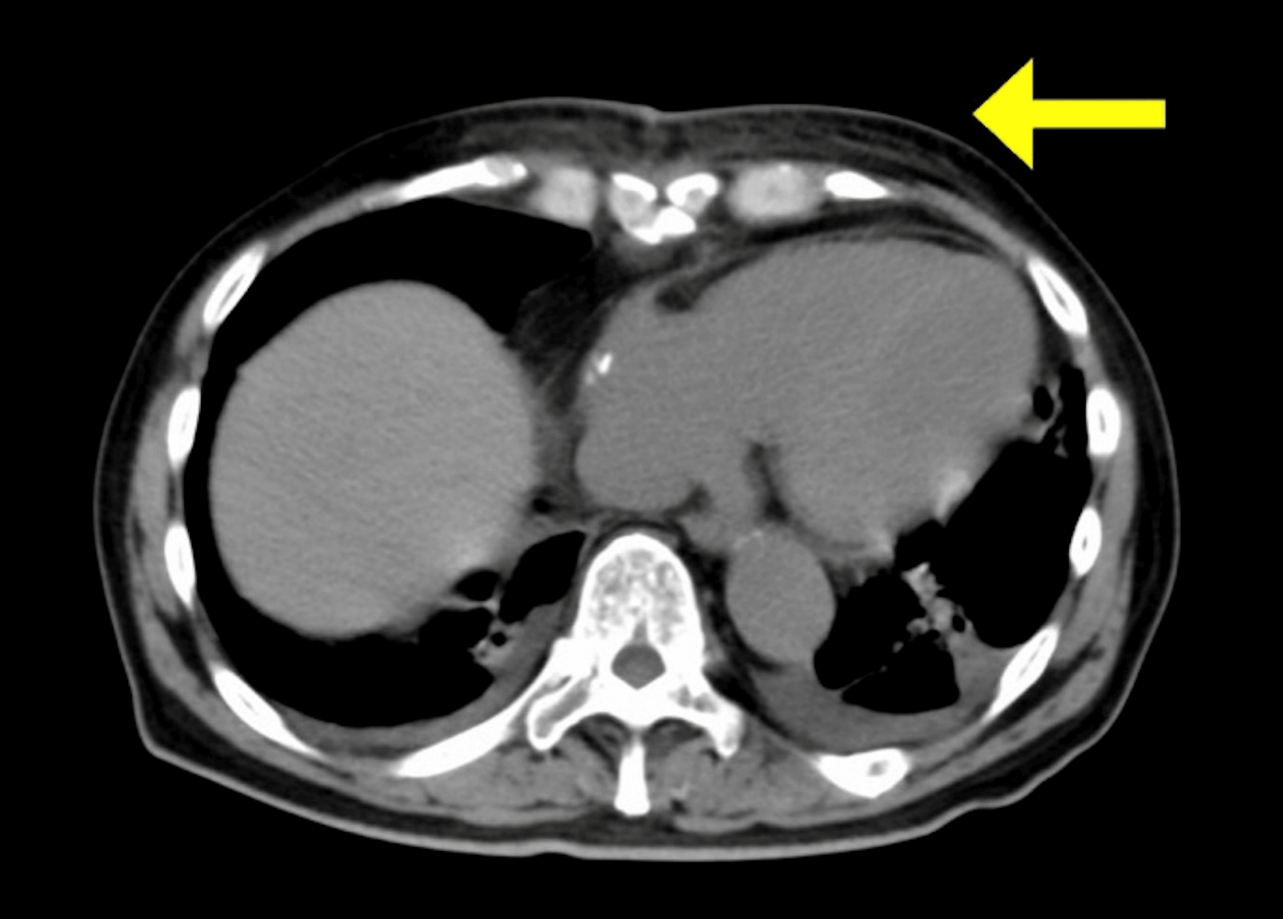
CT on postoperative day 11 A mild increase in fat tissue density is be observed anterior to the sternum

The initial postoperative course was uneventful. The patient was extubated on postoperative day (POD) five, ambulated on POD six, and transferred from the ICU on POD eight. However, on POD 11, he developed a fever of 38.9 °C, and erythema was present around the surgical wound (Figure 1). CT revealed a slightly increased density in the presternal fat, with no evidence of seroma enlargement or sternal dehiscence (Figure 2). Blood tests revealed elevated levels of inflammatory markers and lymphopenia (Table 1). Ampicillin-sulbactam was initiated empirically and switched to meropenem on POD 12 due to mediastinitis.

**Table 1:** Laboratory investigations ALT - alanine aminotransferase; AST - aspartate aminotransferase; CRP - C-reactive protein

Parameters	POD 11	Reference values
Total leukocytes (x10/uL)	9.3	4.0-10.0
Hematocrit (%)	33.7	40-50
Hemoglobin (gm/dL)	11.6	13-17
Platelet (x103/uL)	324	150-410
Serum urea (mmol/L)	7.8	2.5-7.8
Serum creatinine (umol/L)	1.21	62-106
Serum potassium K (mmol/L)	3.9	3.5-5.3
Serum sodium (mmol/L)	134	133-146
ALT (IU/L)	75	0-41
AST (IU/L)	45	0-41
CRP (mg/L)	21.74	0-5

**Figure 3:**
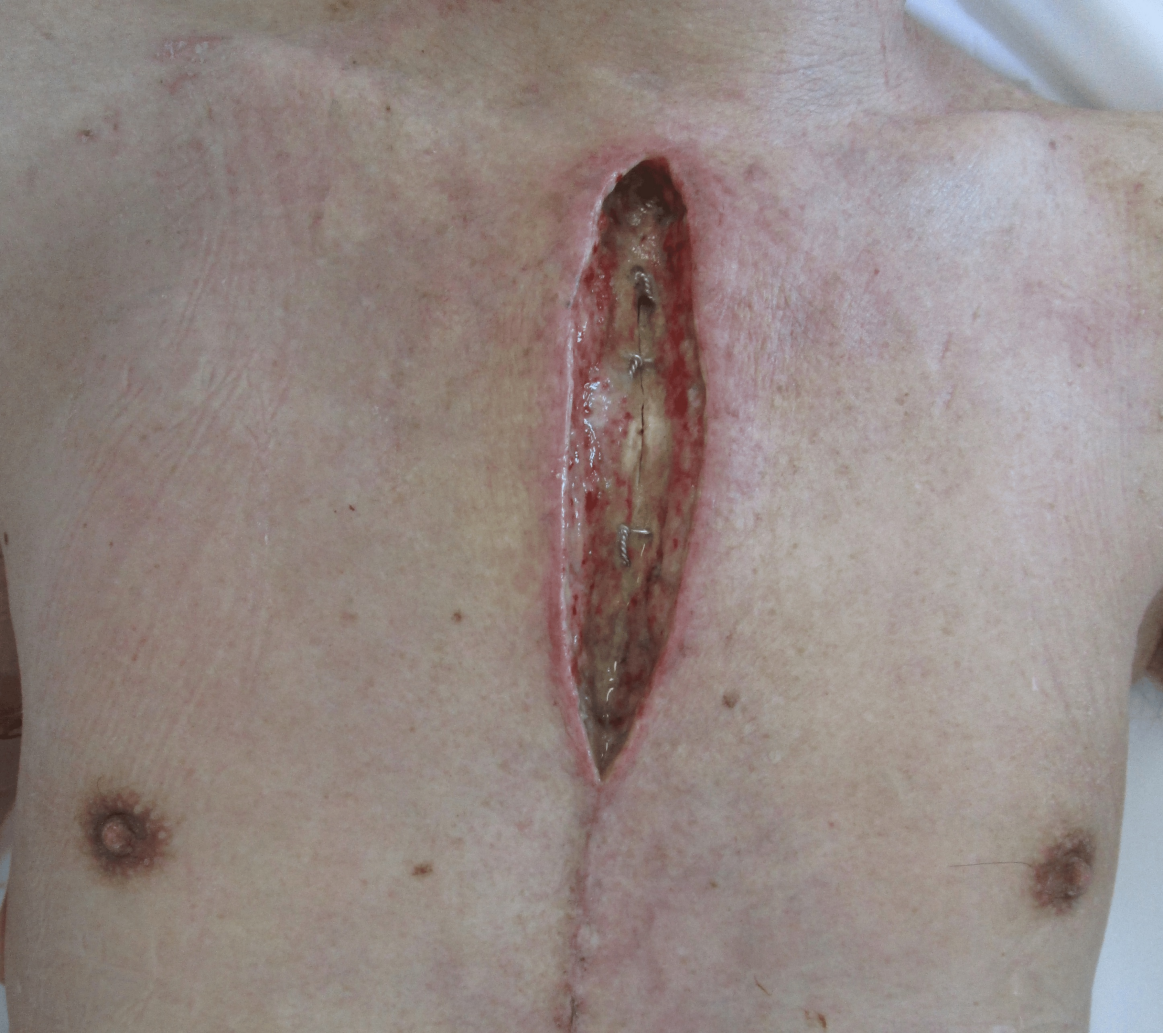
Wound appearance on postoperative day 16 (after incision) The surgically incised and debrided wound reveals exposure of the sternal wire

On POD13, surgical debridement and drainage of the anterior chest wound were performed (Figure 3). CT on POD14 showed no signs of deep abscess or sternal instability. Blood and wound cultures revealed the presence of MRSP, which was identified using matrix-assisted laser desorption/ionization time-of-flight (MALDI-TOF) mass spectrometry. Upon further history taking, it was newly revealed that the patient had a pet dog. Antimicrobial susceptibility testing demonstrated that the isolate was susceptible exclusively to vancomycin, minocycline, and teicoplanin, with resistance observed against all other antimicrobial agents tested. Based on these results, treatment was de-escalated to vancomycin on POD 15. Starting on POD 16, vacuum-assisted closure (VAC) therapy was initiated and repeated every three to four days for a total of four sessions. The wound was well-granulated, and VAC therapy was discontinued on POD 39. Subsequently, the wound was treated with topical prostaglandins and trafermin.

**Figure 4:**
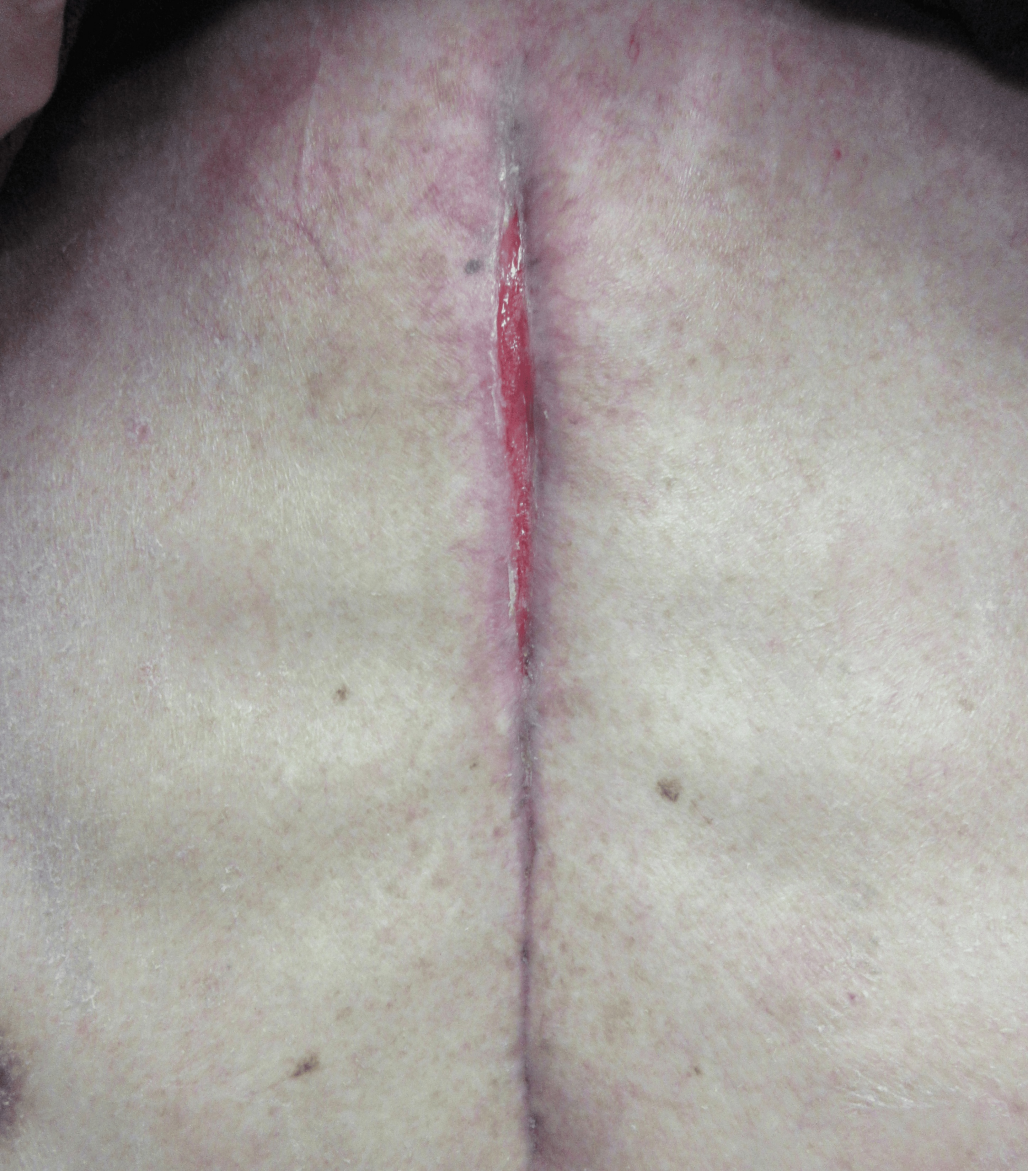
Wound appearance on postoperative day 58 (after healing) Well-developed granulation tissue can be observed, and the wound is completely closed.

Follow-up CT on POD22 and POD39 showed no mediastinal abscess or sternal dehiscence. Due to pancytopenia and a drug-induced rash, vancomycin was switched to teicoplanin on POD32, which was discontinued the following day, and inflammatory markers on blood testing demonstrated a marked improvement, with the C-reactive protein (CRP) level having decreased to 2.5 mg/dL. As MRSP is a known commensal of canine skin and mucosa, and the patient owned a dog, he was advised to avoid close contact with the animal after discharge. On POD 44, the patient was discharged home, and the wound was completely closed on POD 53 (Figure 4).

## Discussion

PSM is among the most severe postoperative infectious complications of cardiovascular surgery, with an incidence of 0.5-2.2% and a mortality rate of 10-30% [1]. Various factors contribute to its onset, including diabetes mellitus, obesity, smoking history, reoperation, prolonged surgical duration, and the use of bilateral internal thoracic arteries [2]. Multifaceted therapeutic strategies are essential for diagnosis and management. The most common causative organisms are skin-residing staphylococci, such as methicillin-resistant *Staphylococcus aureus* and *Staphylococcus epidermidis*. Standard treatment involves a multidisciplinary approach combining antibiotic therapy, surgical debridement and drainage, irrigation, and when necessary, reoperation or VAC therapy [3,4].

In particular, VAC therapy exerts multiple wound-healing effects by applying continuous negative pressure to the wound, which facilitates exudate removal, improves local perfusion, promotes granulation tissue formation, and reduces bacterial load [5]. In recent years, it has gained widespread adoption as an effective therapeutic option for PSM. According to Vos et al., VAC therapy significantly reduces mortality and recurrence rates compared to conventional open wound management, and is especially advantageous in high-risk patients with poor general condition [6]. Moreover, VAC therapy serves as a bridging therapy until definitive reconstruction with musculocutaneous flaps, enabling concurrent infection control and wound-bed optimization. Therefore, VAC therapy is no longer limited to local wound management but has been incorporated into the standard PSM management strategy as a comprehensive infection control measure, and it proved effective in the present case.

A notable aspect of the present case was the identification of *S. pseudintermedius*, a coagulase-positive *Staphylococcus* commonly found on canine skin, as the causative organism of PSM in its methicillin-resistant form (MRSP). While well-recognized in veterinary medicine as a common cause of pyoderma, otitis externa, and wound infections in dogs, human infections by this organism are rare and usually occur in immunocompromised patients, individuals with indwelling devices, or those in close contact with animals [7].

Although the patient in this case owned a dog, there was no direct contact with the animal during postoperative hospitalization. This implies that the infection may not have resulted from direct transmission, but rather from prior colonization of the patient's skin with MRSP. *S. pseudintermedius* has been shown to transiently colonize humans through contact with dogs, often resulting in asymptomatic carriage. However, under conditions such as surgical trauma or temporary immunosuppression, MRSP colonized on the skin may act as a pathogen, leading to postoperative infection [8]. Given its multidrug-resistant nature, MRSP poses therapeutic challenges and requires careful consideration for infection control. On the other hand, it has been reported that Staphylococcus pseudintermedius is often misidentified as *Staphylococcus aureus* by conventional morphological methods [9]. Accurate identification of the organism requires access to advanced diagnostic capabilities, such as polymerase chain reaction (PCR) testing, and this should be taken into consideration. Reports of MRSP as a causative agent of PSM after cardiac surgery are exceedingly rare, making this an unusual case.

This case highlights the importance of considering zoonotic pathogens as potential causative agents of postoperative infections. Given the increasing rate of pet ownership, even in urban settings, contact history with household animals should not be overlooked during preoperative assessments and infection risk stratification. The MAR study recommends that, in the postoperative setting, high-risk patients such as the elderly and immunocompromised individuals should restrict contact with dogs, given the elevated risk of infection [10]. In this case, the patient's pet ownership was revealed only after infection became evident. Therefore, it is imperative that preoperative interviews include detailed inquiries regarding pet ownership and that this information is incorporated into postoperative wound care planning as appropriate. Furthermore, in high-risk populations, such as immunocompromised or elderly patients, screening for *Staphylococcus pseudintermedius* colonization may be warranted to mitigate the risk of postoperative infections.

## Conclusions

We report a rare case of PSM caused by MRSP following ascending aortic replacement. The infection was successfully treated with surgical drainage, targeted antimicrobial therapy, and VAC therapy. This case emphasizes the importance of considering zoonotic pathogens in postoperative infections and incorporating pet contact history in the preoperative evaluation of patients undergoing cardiac surgery.

## References

[1] Gårdlund B, Bitkover CY, Vaage J (2002). Postoperative mediastinitis in cardiac surgery - microbiology and pathogenesis. Eur J Cardiothorac Surg.

[2] Goh SS (2017). Post-sternotomy mediastinitis in the modern era. J Card Surg.

[3] Gårdlund B, Bitkover CY, Vaage J (2002). Postoperative mediastinitis in cardiac surgery - microbiology and pathogenesis. Eur J Cardiothorac Surg.

[4] Sjögren J, Malmsjö M, Gustafsson R, Ingemansson R (2006). Poststernotomy mediastinitis: a review of conventional surgical treatments, vacuum-assisted closure therapy and presentation of the Lund University Hospital mediastinitis algorithm. Eur J Cardiothorac Surg.

[5] Fleck T, Gustafsson R, Harding K (2006). The management of deep sternal wound infections using vacuum assisted closure (V.A.C.) therapy. Int Wound J.

[6] Vos RJ, Yilmaz A, Sonker U, Kelder JC, Kloppenburg GT (2012). Vacuum-assisted closure of post-sternotomy mediastinitis as compared to open packing. Interact Cardiovasc Thorac Surg.

[7] Moses IB, Santos FF, Gales AC (2023). Human colonization and infection by Staphylococcus pseudintermedius: an emerging and underestimated zoonotic pathogen. Microorganisms.

[8] Somayaji R, Priyantha MA, Rubin JE, Church D (2016). Human infections due to Staphylococcus pseudintermedius, an emerging zoonosis of canine origin: report of 24 cases. Diagn Microbiol Infect Dis.

[9] Börjesson S, Gómez-Sanz E, Ekström K, Torres C, Grönlund U (2015). Staphylococcus pseudintermedius can be misdiagnosed as Staphylococcus aureus in humans with dog bite wounds. Eur J Clin Microbiol Infect Dis.

[10] Priyantha Priyantha, M. M. (2022). An overview of human infections caused by Staphylococcus pseudintermedius: a zoonotic risk of the oldest friend. Sri Lankan J Infect Dis.

